# Fourier Transform Infrared (FTIR) Spectroscopy to Analyse Human Blood over the Last 20 Years: A Review towards Lab-on-a-Chip Devices

**DOI:** 10.3390/mi13020187

**Published:** 2022-01-26

**Authors:** Ahmed Fadlelmoula, Diana Pinho, Vitor Hugo Carvalho, Susana O. Catarino, Graça Minas

**Affiliations:** 1 Center for MicroElectromechanical Systems (CMEMS-UMinho), University of Minho, 4800-058 Guimaraes, Portugal; id9247@alunos.uminho.pt (A.F.); scatarino@dei.uminho.pt (S.O.C.); 2 LABBELS–Associate Laboratory, Braga/Guimaraes, Portugal; 3 International Iberian Nanotechnology Laboratory (INL), 4715-330 Braga, Portugal; diana.pinho@inl.int; 4 Algoritmi Research Center, University of Minho, 4800-058 Guimaraes, Portugal; vcarvalho@ipca.pt; 5 2Ai, School of Technology, IPCA, 4750-810 Barcelos, Portugal

**Keywords:** blood cells, fourier transform infrared (FTIR) spectroscopy, functional group, lab-on-a-chip

## Abstract

Since microorganisms are evolving rapidly, there is a growing need for a new, fast, and precise technique to analyse blood samples and distinguish healthy from pathological samples. Fourier Transform Infrared (FTIR) spectroscopy can provide information related to the biochemical composition and how it changes when a pathological state arises. FTIR spectroscopy has undergone rapid development over the last decades with a promise of easier, faster, and more impartial diagnoses within the biomedical field. However, thus far only a limited number of studies have addressed the use of FTIR spectroscopy in this field. This paper describes the main concepts related to FTIR and presents the latest research focusing on FTIR spectroscopy technology and its integration in lab-on-a-chip devices and their applications in the biological field. This review presents the potential use of FTIR to distinguish between healthy and pathological samples, with examples of early cancer detection, human immunodeficiency virus (HIV) detection, and routine blood analysis, among others. Finally, the study also reflects on the features of FTIR technology that can be applied in a lab-on-a-chip format and further developed for small healthcare devices that can be used for point-of-care monitoring purposes. To the best of the authors’ knowledge, no other published study has reviewed these topics. Therefore, this analysis and its results will fill this research gap.

## 1. Introduction

Millions of blood test analyses are performed every day worldwide in order to provide blood diagnostic services for the patients [1]. Usually, these tests are performed in clinical laboratories, simultaneously using different devices and relying on different specialties [2]. These devices are needed to run routine blood tests [2] and examine multiple parameters to assist the physicians in haematology-, chemistry-, and immunology-related diagnosis, among others. They require human resources, dedicated facilities, and time, which, in an ideal device, should be less than one hour from taking a sample to printing out the results [3,4]. Moreover, the reagents needed to run all these tests are expensive, and most of them are toxic, having a significant direct and indirect effect on the environment [5].

Diagnostic devices currently available on the market rely on the same measuring techniques developed in the last century (mainly spectrophotometry or electrochemical assays) [6]. Meanwhile, viruses, bacteria, and fungi are rapidly evolving [7], pushing further the need to develop new, quick, and reliable diagnostic tools. The primary, commercially available measuring techniques for such devices are spectrophotometry, enzyme-linked immunosorbent assay (ELISA), electrophoresis, and blood cell counting or complete blood count (CBC). However, all of these methods have limitations. In ultraviolet–visible (UV–VIS) spectrophotometry, the main limitation is the requirement for sample and setup preparation time to avoid light interferences [8]. ELISA limitations are related to the cost of the assays due to the use of antibodies, the risk of cross-reactivity, the high background noise, and extended analysis time [9]. Electrophoresis requires a large sample for the assays, as well as high analysis precision [10]. Finally, CBC limitations are related to the manual examination of blood smears, difficulty recognising abnormal red cell shapes (such as fragmented cells), and high running costs [11]. Hence, the pressing need for new, fast, and precise analysing techniques.

Fourier transform infrared (FTIR) spectroscopy is a field that has undergone significant development over the past decade, promising easier, more rapid, and more objective diagnoses [12,13]. FTIR spectroscopy studies the interactions between matter and electromagnetic radiation that appear in the form of a spectrum. Each molecule has a spectrum fingerprint that makes it unique and allows it to be distinguished from other molecules [14]. FTIR spectroscopy is also an effective and nondestructive method for monitoring cellular alterations [15,16]. FTIR spectral analysis has allowed the characterisation of several organs’ diseases, as well as the quantification of different biomolecules such as proteins [16], nucleic acids [17,18], and lipids [19]. Several research documents highlighting the importance of spectroscopic techniques in cancer detection have been published in the literature [15,20,21]. FTIR focuses on the differentiation and characterisation of cells and tissues by looking at individual bands or groups to precisely identify the molecular conformations, bonding types, functional groups, and intermolecular interactions that compose the specimen [13,20]. Thus, this paper describes the main concepts and terminologies related to FTIR and presents the latest published research focusing on FTIR spectroscopy technology and its integration in lab-on-a-chip devices for application in the biological field. To the best of the authors’ knowledge, no other studies have reviewed these topics, making this review the first to fill this research gap.

The paper is organised into five sections. Section 1 presents an introductory overview and the primary motivation guiding the study. Section 2 presents FTIR spectroscopy’s theoretical, conceptual elements and clarifies the salient terminology, including the concepts of infrared (IR) regions, radiation, molecular vibration, FTIR, and Michelson Interferometer. Section 3 describes the methods used in the presented study, while Section 4 offers the results of the analysis of twenty-year-long research on the application of FTIR spectroscopy in the biological field, focusing on the possibility of applying this technology in lab-on-a-chip devices. Finally, Section 5 presents the conclusion and future trends.

## 2. Theoretical Considerations

Here the terminologies and concepts associated with FTIR spectroscopy, namely the IR region, IR radiation and molecular vibrations in biological matters, FTIR techniques and Michelson interferometer, are presented.

### 2.1. Infrared Region

**Figure 1:**
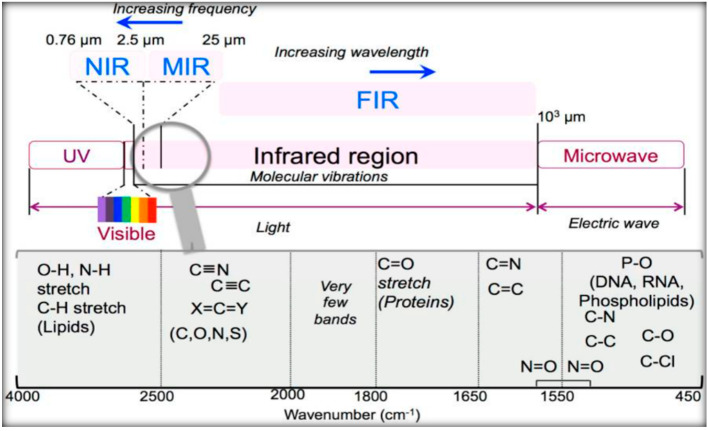
Scheme of the optical spectrum, focusing on the infrared region. Reprinted from [23], MDPI, under a Creative Commons Attribution (CC BY) license.

IR radiation is a group of electromagnetic waves (EMR) with wavelengths longer than visible radiation, invisible to the human eye. The IR region of the electromagnetic spectrum ranges in wavelengths from 0.8–100 µm, illustrated in Table 1 [22,23]. Typically, the IR is broken into three ranges, near-IR, mid-IR, and far-IR. Most of the IR used in medical applications are in the mid-IR range, considering radiation from the electromagnetic spectrum, in the wavenumber interval from 4000 cm^−1^ to 400 cm^−1^. The frequency of the absorbed radiation is responsible for each subatomic vibrational interaction, as schematised in Figure 1.

### 2.2. IR Radiation and Molecular Vibrations in Biological Matter

As a type of electromagnetic wave, IR propagates energy and momentum, with properties similar to both a wave and a particle—the photon. IR is emitted or absorbed by molecules as they change their rotational, vibrational motions. It excites wave modes in a molecule by changing them instantaneously, making it a helpful frequency variation for studying the molecular energy states with correct symmetry. Therefore, IR chemical analysis studies the absorption and transmission of photons in the IR region [24]. The IR spectrum of biological samples consists of a combination of the characteristic absorption bands of proteins, lipids, nucleic acids, and carbohydrates within that sample [25,26].

The protein absorption bands are often assigned to amino acid side groups or peptide backbone in the 1700 cm^−1^–1500 cm^−1^ range. The vibrational modes of the peptide backbone produce the amide I and II bands. The amide I band (1700 cm^−1^–1600 cm^−1^) is mainly associated with the bending vibration of the N–H bond. The bands of amides I and II are usually used to analyse the secondary protein structure [27]. The presence of absorption bands at 1450 cm^−1^ and 1400 cm^−1^ is due to asymmetric and symmetric methyl bending modes, respectively [28].

In the spectra of lipids, absorption bands are found in numerous spectral regions: the range of 3050 cm^−1^–2800 cm^−1^ for asymmetric and symmetric stretching vibrations of -CH2 and -CH3, the range of 1500 cm^−1^–1350 cm^−1^ for deformation vibrations of -CH2 and -CH3 from the acyl chains of lipids, the range of 1745 cm^−1^–1725 cm^−1^ for symmetric stretching vibrations of ester–carbonyl bond (C=O), and the range of 1270 cm^−1^–1000 cm^−1^ for odd (1240 cm^−1^) and symmetric (1080 cm^−1^) vibrations of -PO2- in phospholipids [29]. The IR spectra of nucleic acids are characterised in four spectral regions: the region of 1780 cm^−1^–1550 cm^−1^ for in-plane vibrations of double bonds of bases, the region of 1550 cm^−1^–1270 cm^−1^ for the deformation vibrations of bases that include the sugar vibrations, the region of 1270 cm^−1^–1000 cm^−1^ for vibrations of -PO2- and, finally, the region of 1000 cm^−1^–780 cm^−1^ for the vibrations of the sugar-phosphate backbone [30]. The carbohydrate spectra contain bands in the following ranges: the region of 3600 cm^−1^–3050 cm^−1^ is assigned to the stretching vibration of O-H, the range of 3050 cm^−1^–2800 cm^−1^ to the stretching vibrations of -CH_3_ and -CH_2_, the region of 1200 cm^−1^–800 cm^−1^ to the stretching vibrations of the C-O/C-C species, and, finally, the 1500 cm^−1^–1200 cm^−1^ relates to the deformational modes of the CH_3_/CH_2_ species [31]. In the blood analysis applications, the spectral bands of 3000 cm^−1^–2800 cm^−1^ are the most relevant ones for analysing red blood cells and platelets, while for the white blood cells, the most relevant band ranges are 513 cm^−1^–1445 cm^−1^. Thus, those targeted cells and corresponding spectral bands are the most used for blood analysis, particularly lab-on-a-chip applications.

### 2.3. Fourier Transform Infrared Spectroscopy (FTIR) Techniques

**Figure 2:**
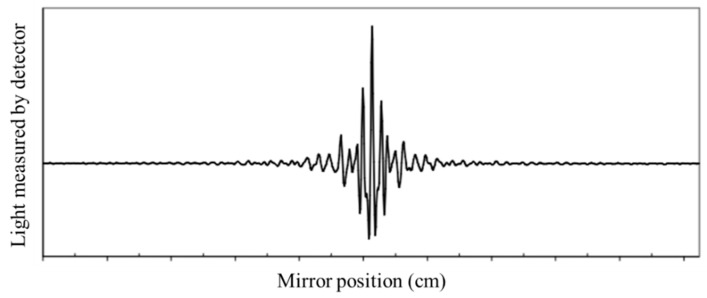
Example of a general FTIR interferogram. The central peak is positioned at the ZPD position (zero path difference or zero retardation), where the maximal amount of light passes through the interferometer to the detector.

FTIR spectroscopy is a technique used to obtain the absorption or emission infrared spectrum of a solid, liquid, or gas [14,32]. The FTIR spectrometer simultaneously collects high-resolution information over a wide spectral range (between 4000 and 400 cm^−1^), a distinct advantage over a dispersive spectrometer, which estimates power over a narrow range of frequencies at once. The aim of spectroscopy techniques (FTIR or bright perceptible (UV–Vis) spectroscopy) is to quantify how much light a sample absorbs at each frequency [14]. The most direct approach, the “dispersive spectroscopy” method, consists of focusing a monochromatic light beam at a sample, measuring the amount of absorbed light, and recalculating it for each frequency [14]. Fourier transform spectroscopy is a less instinctive approach for obtaining similar data. Rather than focusing a monochromatic (single frequency) light emission at the sample, this strategy might focus a bar, or array, which contains numerous frequencies of light at once and measures how much of that beam is absorbed by the sample. Then, the wave is changed to contain a different mixture of frequencies giving a second data point. This cycle is repeated many times within a short period of time, and the information is acquired by a computer. For instance, the wave plotted in Figure 2, called an interferogram, is created by applying a broadband light source-one that contains the entire range of frequencies to be estimated. The light sparks into a Michelson interferometer (detailed in the next section), consisting of a special array of mirrors, one of which is moved by a motor. As this mirror moves, each light frequency in the column is occasionally obstructed, mediated, impeded, and transmitted by the interferometer. The different frequencies are tweaked at different rates so that the column exiting the interferometer has a different range at each second or mirror position [14,32].

Computational postprocessing based on Fourier frequencies is required to calculate the results (light pickup for each frequency) from the coarse raw information (light pickup for each mirror position), as presented in the example of Figure 2 [14,32]. Then, the Fourier transform converts a space (for this situation, the mirror’s distance in cm) into its opposite space (wavenumbers in cm^−1^).

The main limitations of FTIR spectroscopy relate to the tissue depth penetration of the infrared light, which only allows biochemical analysis of the tissues up to a few dozens of micrometres [20]. Additionally, in the conventional FTIR spectroscopy, which works in transmission mode and consequently with no incidence angle between emitter and sample, there is difficulty in assuring the reproducibility of the spacer thickness when using liquid samples [33].

The attenuated total reflectance Fourier transform (ATR -FTIR) technique as a complementary technique has helped FTIR spectroscopy overcome this limitation. ATR-FTIR is a particular FTIR spectroscopy method, which measures the reflected signal from a sample. In this reflectance setup, the IR radiation passes through a crystal with a high refractive index (typically with an angle of 45°) and undergoes total internal reflection before exiting the crystal and being directed to an IR detector [33,34]. ATR-FTIR has a lower penetration depth than conventional FTIR (around 200 nm) but, since it measures the reflected light, it is an adequate method for measuring high absorbing and high thickness samples that typically do not allow the transmission of IR radiation [33]. Additionally, this technique can direct measurements of gas, fluidic and thin-film solid-state samples without complex sample preparation and with enhanced surface sensitivity [33,34].

Finally, microscopic FTIR (micro-FTIR) [35] relates to another particular FTIR technique that couples an IR spectrometer to a visible light microscope in order to achieve better sensitivity when detecting condensed-phase compounds [36] and is adequate for measuring solid or liquid thin films samples. In this technique, the microscope focuses the IR laser beam on the sample, and the measurement mainly comes from the target focal point, meaning that even a short displacement in the laser beam or the sample could provide a significant difference in the results. Therefore, micro-FTIR distinguishes by allowing local measuring of a particular point in the sample, while the conventional FTIR gives the average information from a complete homogenised sample [35,36].

Besides helping to identify organic compounds based on their specific IR spectral fingerprint, FTIR also has a relevant role in detecting alterations or pathological states of the molecules and samples, leading to different spectra between patients and healthy controls, as presented in several examples in Section 4. In the presence of pathology, the IR spectrum of a sample will change, either by changing its intensity or shifting its peak frequencies [37]. These shifts can be due to multiple chemical alterations in the molecules’ composition, including weakening of the bonds, decreasing mass of the molecules, or even shifting the stretching vibrations due to temperature variations, which will change the vibrational frequencies of the bands. More details on this can be found elsewhere [37].

### 2.4. Michelson Interferometer

**Figure 3:**
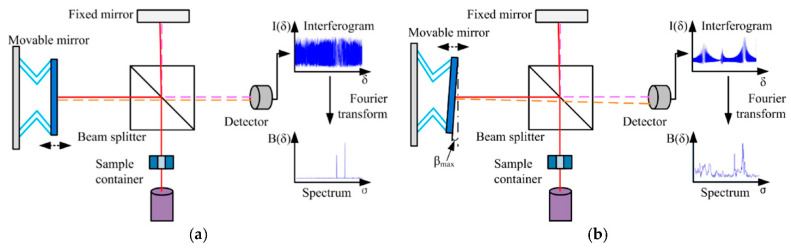
Schematic diagram of a Michelson interferometer configured for FTIR. (**a**) An ideal Michelson interferometer; (**b**) a Michelson interferometer with the movable mirror tilting. The continuous and dashed lines represent the different directions of light. Reprinted from [32], MDPI, under a Creative Commons Attribution (CC BY) license.

The Michelson Interferometer technique was adapted for FTIR so that the light from the polychromatic IR source, effectively a blackbody radiator, is collimated and directed onto a beam splitter, with 50% of the photons by the fixed mirror and 50% transmitted by the movable mirror [32]. In this configuration, light is reflected from the two mirrors back to the beam splitter, and some fraction of the original light passes into the sample compartment Figure 3.

There, the light is focused on the sample. When leaving the sample compartment, the light is refocused on the detector. The difference in the optical path length between the two mirrors to the interferometer is known as the retardation or optical path difference (OPD) [32]. An interferogram (as in Figure 2) is obtained by varying the retardation and recording the signal from the detector for different retardation values. When no sample is present, the interferogram profile depends on the variation of the source intensity and splitter efficiency with wavelength. This results in a maximum at zero retardation when there is constructive interference at all wavelengths, followed by a series of wiggles [32]. This problem is critical in the case of zero default when there is constructive interference within the smallest wavelengths followed by a series of wigglers. The location of the null default is determined by locating the purpose of the excessive intensity within the interferogram. When a pattern is given away, the course interferogram is modulated with the aid of the absorption bands within the pattern (as exemplified in Figure 3) [32].

## 3. Methods

The research and data collecting strategy was based on evaluating a wide scale of papers (matching the topic keywords) published in the FTIR spectroscopy field, in the last decades, with all adequate reference and copyright permissions. For that, a comprehensive electronic search on ScienceDirect, Scopus and PubMed databases was performed (up to October 2021, Q3), as well as a direct search on different publishers’ specific databases, such as MDPI, Wiley, or Nature, among others. Search keywords included: FTIR, spectroscopy, optics, infrared, blood, blood cells, Functional Group, Michelson Interferometer, lab-on-a-chip, microfluidics, microdevice and diagnostics. The search strategy was established by combining several keywords and using AND/OR Boolean operators. The relevant studies resulting from the database search were manually analysed to identify other potential studies to be included. The exclusion criteria were: reviews, comments, overviews, case reports, viewpoints and perspectives, as well as documents reporting tests with data ambiguity. Studies not written in the English language were also excluded, as well as duplicate results. From there, titles and abstracts were screened. All abstracts were read, and those that did not fit the purpose of this review were excluded. The information regarding the application, quantitative outcomes, reported study limitations, and other relevant comments were selected and extracted from the remaining articles. Specifically, the authors selected papers that reported FTIR spectroscopy for analysing between normal blood samples and pathological blood samples for cancer detection, HIV early recognition in pregnant women, and blood grouping identification, among others. These applications were chosen to illustrate the wide range of FTIR applications. Finally, the most important conclusions and limitations on the analysed papers were summarised.

## 4. Results

This section presents the data collection results examining the most relevant studies over the last twenty years addressing FTIR in the biological field, organised by their application field.

### 4.1. Collection of Data Seeking Applications of FTIR Spectroscopy

**Figure 4:**
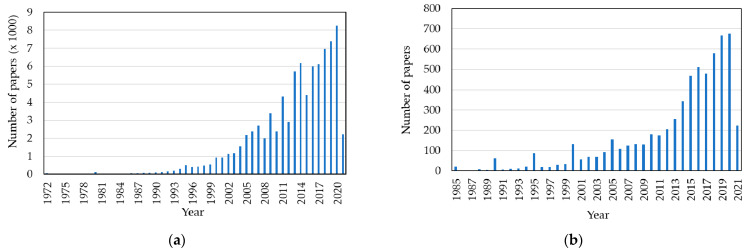
Published papers focusing FTIR: (**a**) Overall papers, since 1972; (**b**) papers in the biological field, since 1985 (until 2021, Q3).

FTIR is widely used as a diagnostic method to analyse different materials and samples. According to the PubMed search results, FTIR spectroscopy was first studied as a new potential method in 1972, and until now (2021, Q3), more than 76,900 papers have been published on FTIR spectroscopy. A few years later, in 1982, researchers in the biological field recognised the potential of the FTIR techniques and their suitability for diagnostics, including diagnoses of a long list of diseases that included cancer, microbes, bacteria, and viruses detection. The number of papers relating to FTIR published in the biological field in 2021 (up to Q3) is around 4810. Figure 4a,b illustrate the evolution of FTIR studies over the last two decades.

**Figure 5:**
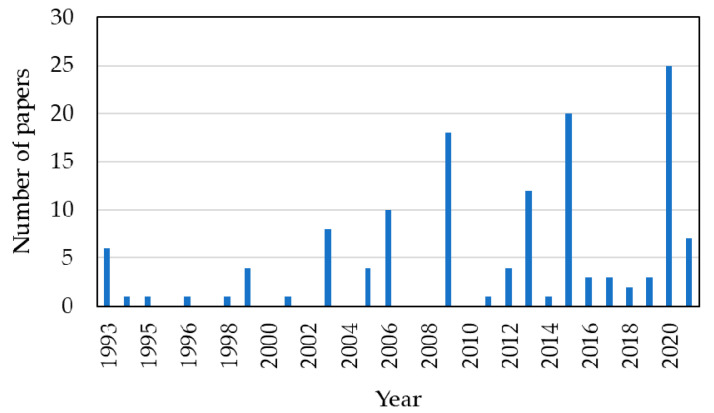
Published papers focusing FTIR for addressing differentiation between normal/pathological blood samples, from 1999 until 2021 (Q3).

In particular, by analysing the PubMed search results, a short number of papers addressed the use of FTIR spectroscopy to analyse and distinguish between normal blood sample cells and pathological blood samples. From the research undertaken until 2021, this number is less than 100 (Figure 5).

**Figure 6:**
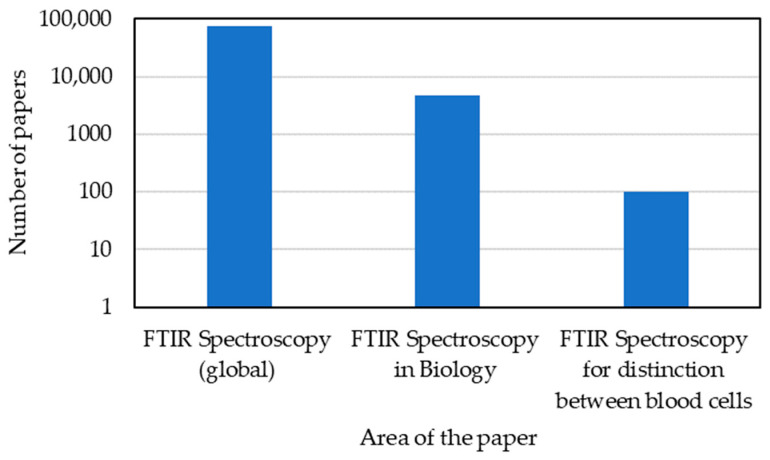
Summary of the total published papers focusing FTIR from 1999 until 2021 (Q3).

Figure 6 summarises the number of published papers considering the FTIR subject (total of 76,900 papers) and its specificity in the application of the biology domain (4810) and subdomain for distinguishing between normal and pathological blood samples (around 100).

### 4.2. Applications of FTIR Spectroscopy in Cancer Diagnosis

Among the different spectroscopic techniques developed to distinguish between normal and cancerous blood tissues, Fourier transformed spectroscopy has shown tremendous potential. Additionally, biomedicine’s IR-based techniques have become a reality with a large amount of information accumulated from clinical studies, trials, and developments [38,39,40].

**Figure 7:**
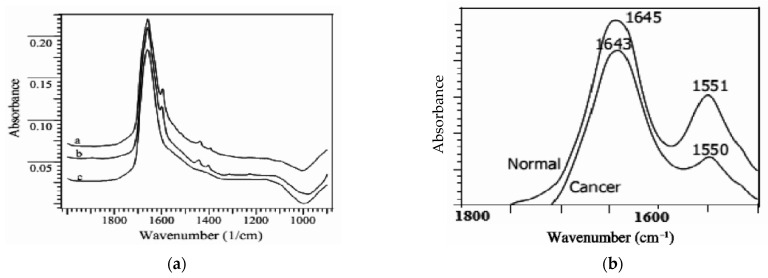
(**a**) FTIR absorption spectra of ‘a’ cancerous blood, ‘b’ normal blood and ‘c’ water samples using air as a reference; (**b**) detail of the FTIR absorption spectra of the normal and cancerous blood. Reprinted from [38], Copyright 2010 Convener, MMSETLSA-2009, with permission from the authors.

In 2013, FTIR spectroscopy was applied to study healthy and cancerous blood samples, using a diffuse reflectance technique from SHIMADZU 8000 series FTIR spectrophotometer. The spectra of cancerous and healthy blood were registered at a resolution of 4 cm^−1^ in the region of 900 cm^−1^ to 2000 cm^−1^, as observed in Figure 7a. The obtained results show that the bands of proteins, lipids, carbohydrates, and nucleic acids from cancerous samples are clearly different from the normal ones dominated by two absorption bands at 1643 cm^−1^–1550 cm^−1^, known as amide I and amide II. Amide I appears from the C=O stretching vibrations and amide II from the C–N stretching and CNH bending vibrations. This wavelength band looks strong and sharp in a healthy blood sample [38], as seen in Figure 7b.

In 2016, label-free FTIR was used for early cancer detection in blood samples. This technique allowed detecting and verifying spectral biomarker candidate patterns to detect non-small cell lung carcinoma (NSCLC). The study was conducted on 161 patients where blood serum and plasma samples were analysed using an automatic FTIR spectroscopic system, together with pattern recognition algorithms, such as Monte Carlo cross-validation, linear discriminant analysis and random forest classification. Marker patterns for cancer discrimination (both from squamous-cell carcinoma and adenocarcinoma patients) from clinically relevant disease control patients were identified in FTIR spectra of blood samples. The analysis was constrained to the respective C–H-stretching in the 2800 cm^−1^ –3200 cm^−1^ region and the fingerprint regions 1750 cm^−1^–875 cm^−1^ [39]. Accuracy of up to 79% was recorded [39]. According to the authors, the study demonstrates the applicability of FTIR spectroscopy using blood for lung cancer detection. Evidence for cancer subtype discrimination was given. With improved performance, the method could be developed as a routine diagnostic tool for blood testing of NSCLC [39].

Another study from 2018 demonstrated that ATR-FTIR spectroscopy is a potential technique that can be used for cutaneous melanoma (i.e., skin cancer) diagnosis and for differentiating the metastatic potential of cancer cells. By using IR spectroscopy, one can identify various types of cancer such as basal cell carcinoma, malignant melanoma, nevus, as well as metastatic potential by evaluating the alterations in hydration level and molecular changes [40]. The spectra obtained by the authors show different intensities and frequencies of normal and cancerous samples in the spectral range between 4000 cm^−1^ and 400 cm^−1^. The region between 4000 cm^−1^ and 3000 cm^−1^ shows stretching vibrations of O-H and N-H corresponding to the spectral bands of collagens and proteins of the skin. As cancer changes, the permeability of the cells’ membrane and the metastatic potential also change with the hydration grade of the cell membrane; the ATR-FTIR spectroscopy is an approach that allows successful differentiation of the metastatic potential of cancer cells [40]. In particular, the comparison between fewer and more metastatic cells shows that the hydration level of the plasma membrane leads to a significant difference between both states of cancer [40].

**Figure 8:**
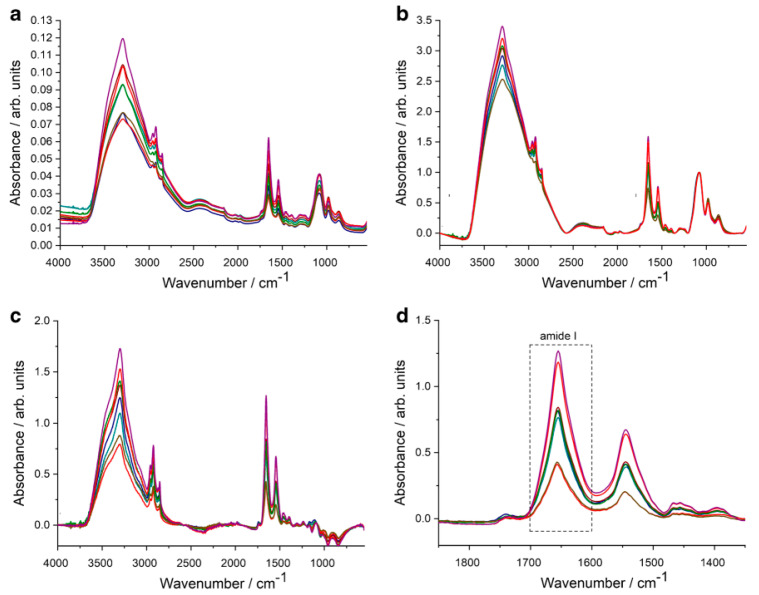
ATR-FTIR spectroscopy quantifies the protein content of extracellular vesicles (EV) samples. (**a**) Raw absorbance spectra after ATR correction. (**b**) Absorbance spectra after baseline correction and normalisation. (**c**) Absorbance spectra after buffer subtraction. (**d**) Zoomed absorbance spectra for calculating area under the curve (AUC) values of the amide I band by integration in 1700 cm^−1^–1600 cm^−1^ wavenumber region. Reprinted from [41], SpringerLink, under a Creative Commons Attribution 4.0 International License.

A study from 2020 presented an easy to use, a reagent-free method based on (ATR-FTIR) spectroscopy to quantify the protein content of extracellular vesicles (EV) samples with no sample preparation [41]. After calibration with bovine serum albumin, the protein concentration of red blood cell-derived EVs (REVs) was investigated by ATR-FTIR spectroscopy. The integrated region of the amide I band was calculated from the IR spectra of REVs, which was proportional to the protein quantity in the sample. Discriminatory protein bands of amide A, amide I and amide II were set at 3298, 1657, and 1546 cm^−1^, respectively. In the reported study, vibrations corresponding to the lipid components were also witnessed as antisymmetric and symmetric methylene stretching of acyl chains in the range 2924 cm^−1^ to 2850 cm^−1^, and the C=O stretching at −1738 cm^−1^ of the glycerol esters, respectively [41], as shown in Figure 8.

This new method presents a reagent-free alternative to traditional colourimetric protein determination assays and requires no special sample preparation to investigate EVs [41]. Therefore, this IR spectroscopy–based protein quantification method can be successfully adapted to the routine analysis of extracellular vesicles.

**Figure 9:**
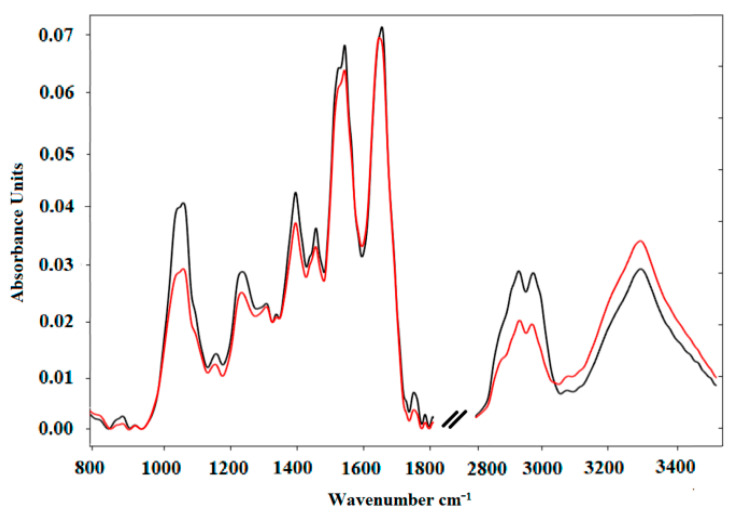
FTIR as a tool for detecting BCP-ALL biomarkers for early screening of pediatric leukaemia. Normalised average FTIR spectra of serum samples: control (black) and Acute Lymphoblastic Leukemia Precursor B (red). The presented spectra cover the range of 800 cm^−1^–3500 cm^−1^. Reprinted from [42], MDPI, under a Creative Commons Attribution (CC BY) license.

FTIR was also used to detect biomarkers for early screening of pediatric leukaemia [42]. In the reported study, the spectra were acquired from blood serum samples of ten child patients with B-cell precursor lymphoblastic leukaemia (BCP-ALL) and were contrasted with ten control samples. No clear peak shift was spotted between the averaged spectra of leukaemia patients and healthy individuals at the first trial. Thus, the authors applied the ratios of particular corrected peaks heights and the second derivatives analytical approaches to better distinguish between BCP-ALL and the control sample. A significant shift was observed for the peak corresponding to the amide I band (1700 cm^−1^ to 1600 cm^−1^) due to the C=O stretch vibrations of the peptide linkages. The frequencies of the amide I band are originally fixed to the secondary structure of the proteins. The position of the amide I band was at 1645 cm^−1^ in the FTIR spectrum of the control group, whereas for the BCP-ALL patients, the peak was shifted to 1641 cm^−1^ [42], as seen in Figure 9.

Thus, the differences between the FTIR spectral profile of leukemic and normal serum may offer a potential route to the early identification of children with BCP-ALL, limiting the number of invasive procedures and accelerating the diagnosis of individuals. The possibility of the early detection of leukaemia in children based only on the FTIR analysis of their serum seems an attractive tool for routine medical practice [42].

### 4.3. Applications of FTIR Spectroscopy in HIV Early Detection

**Figure 10:**
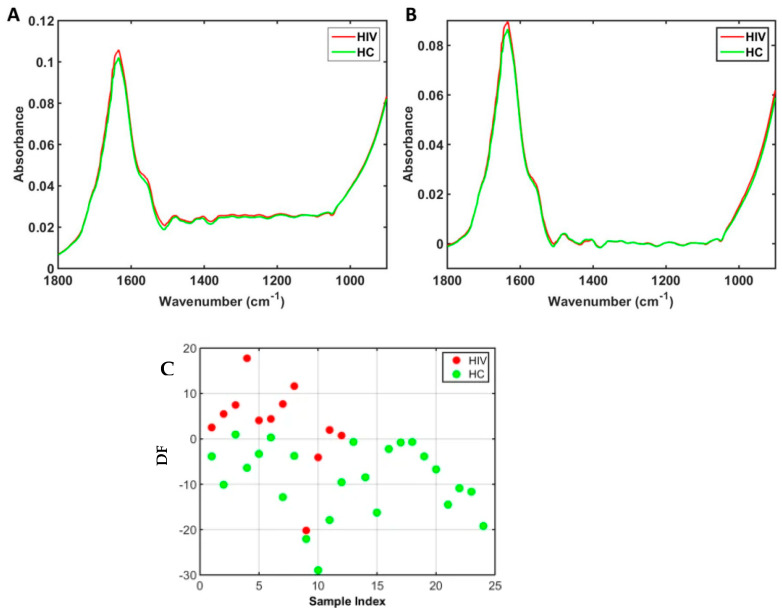
ATR-FTIR spectra for distinguishing between HIV infected and healthy blood samples. (**A**) Mean raw IR spectra in the biofingerprint region (1800 cm^−1^–900 cm^−1^) for HIV-infected (HIV) and healthy uninfected controls (HC) samples. (**B**) Mean preprocessed IR spectra (AWLS baseline correction) in the biofingerprint region (1800 cm^−1^–900 cm^−1^) for HIV-infected (HIV) and healthy uninfected controls (HC) samples. (**C**) Discriminant function (DF) for the samples in the test set, where HIV stands for HIV-infected samples and HC for healthy uninfected controls, allowing their distinction. Reprinted from [43], Nature, under a Creative Commons Attribution 4.0 International License.

In 2020, ATR-FTIR spectroscopy was considered for distinguishing HIV-infected patients from healthy uninfected controls [43]. This study comprised one hundred and twenty blood plasma samples of pregnant women and allowed to obtain good sensitivity (83%) and specificity (95%) using a genetic set of rules with linear discriminant assessment (GA-LDA). In the range of 1800 cm^−1^ to 900 cm^−1^, the spectra displayed some particular feature absorptions, including the amide I band at 1635 cm^−1^, an arm at 1560 cm^−1^ (due to C=O, Amide II) and three small depth absorptions at 1480 cm^−1^ (corresponding to the C-H asymmetric deformation of methyl agencies), at 1404 cm^−1^ (due to the COO−symmetric stretching of proteins and lipids) and 1060 cm^−1^ (due to the C-O nucleic acids). Due to the similarity between the spectral features in the groups (uninfected control and HIV infected), chemometric patterns were used to identify spectral features responsible for class differentiation Figure 10. ATR-FTIR spectroscopy with multivariate analysis was able to accurately identify HIV-infected pregnant women based on blood plasma, showing the potential of this method for early detection of HIV in a fast and reagent-free approach. Successful development of this method in a clinical environment could aid early diagnosis of gestational HIV and help treatment [43].

### 4.4. Applications of FTIR Spectroscopy in Blood Grouping Analysis

In 2017, a study explored the potential for the spectroscopic identification of blood antigens using an FTIR spectrophotometer (Shimadzu FTIR-8400S) within the range of 4000 cm^−1^ to 400 cm^−1^ [44]. The ABO blood type system is reflected in the FTIR spectra of human blood. Specific bands at 1166 cm^−1^ and 1020 cm^−1^ represent the fucose molecules linked glycosidically with galactose and -GlcNAc-, respectively, related to the O antigen.

When -GalNAc- is linked to -O antigen- through glycoside linkage, it exhibits a band at 1022 cm^−1^, due to the -A antigen-. A band at 1166 cm^−1^ reveals additional galactose glycosidically bonded to -O antigen-, as seen in Table 2. Summarily, the IR spectroscopic data on human blood of groups A, B, AB, and O explores the possibility of the nonlabelled and reagent free identification of blood antigens using FTIR [44].

### 4.5. Applications of FTIR Spectroscopy in Blood Analysis

FTIR spectroscopy has also been considered in human blood analysis [13]. In 2004, a study presented a novel methodology for predicting the health status using FTIR-MC (micro-spectroscopy) data on blood components. In this study, FTIR-MC was complemented by cluster analysis algorithms (i.e., the task of grouping a set of objects in such a way that objects in the same group are more similar to each other than to those in other groups) [45]. The FTIR microscopic spectra of the major blood components, which include white blood cells (WBCs), red blood cells (RBCs), and plasma, were isolated from ten controls (average population). All the spectra were normalised to the amide I peak at 1643 cm^−1^.

**Figure 11:**
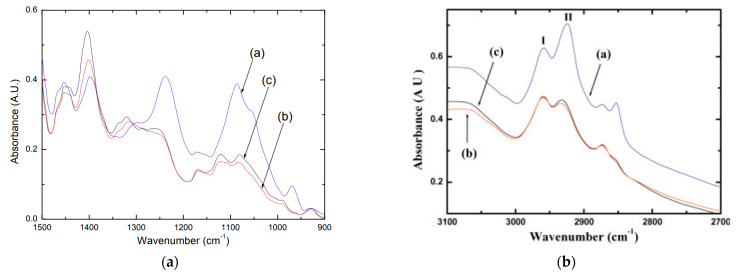
FTIR spectra of the major blood components: WBCs, RBCs and plasma, aiming for blood analysis. (**a**) Expanded region of FTIR-MC spectra (900–1500 cm^−1^) displaying the spectral differences in the symmetric and asymmetric stretching regions of the phosphate group, obtained by the average of ten representative controls; (**b**) FTIR-MSP spectra of the blood components of the averages of 10 representative controls in the 2700–3100 cm^−1^ region. (a) WBCs (blue); (b) RBCs (red); (c) Plasma (black) [45]. Adapted from [45] with permission from Wiley.

The results reported by the authors showed that there are spectral variations between the three blood components to evaluate the validity of the method. Cluster analysis of the WBCs spectra in the 945 cm^−1^ to 1282 cm^−1^ range (comprises both symmetric and asymmetric regions of phosphate) and, more particularly, in the more specific range, from 1146 cm^−1^ to 1282 cm^−1^, provided similar results, as shown in Figure 11a,b. The predictions (FTIR has also been used to analyse the body fluids for diagnostic and characterisation) matched the physician’s diagnosis with 100% accuracy, proving the FTIR-MC as a potential tool to predict the health status of blood samples [45].

Summarily, FTIR-MC can distinguish between the three main components of blood using spectral variations and cluster analysis. Specific spectral changes were observed between infected patients and age-matched healthy controls, providing good classification [45].

### 4.6. Other Applications of FTIR Spectroscopy in the Biological Field

Table 3 presents applications for the FTIR in the biological field tackled rather than blood cell distinction. As observed, FTIR is widely used in biology applications due to its potential to distinguish between different types of molecules.

### 4.7. Applications of FTIR Spectroscopy Integrated with Lab-on-a-Chip Devices

**Figure 12:**
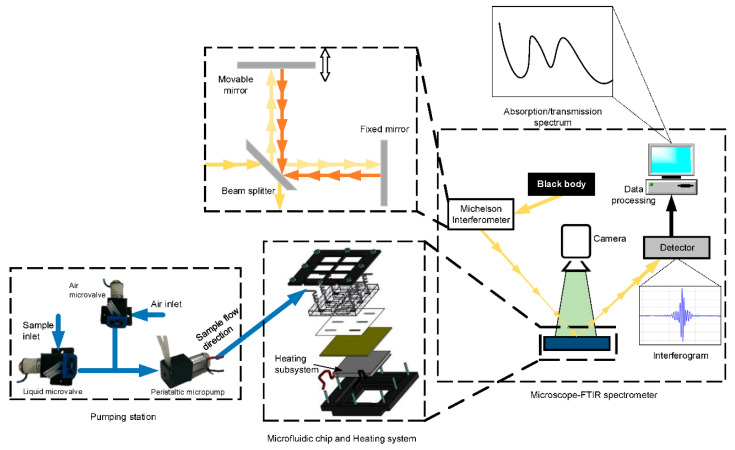
Schematic of the working principle of a pseudo-continuous flow FTIR system, integrated on a microfluidic device for sugar identification. The system includes a pumping station, a microfluidic device, a heating system (for temperature control), and a microscope-FTIR spectrometer. Reprinted from [61], MDPI, under a Creative Commons Attribution (CC BY) license.

Besides the macroscale FTIR applications, it has also been introduced into many modern technologies. In particular, lab-on-a-chip is a technology that has revolutionised and continues to revolutionise the medical field [60]. It intends to convert health care equipment into small devices that can be applied as point-of-care (PoC) methods for monitoring proposes. There are several examples in the literature of the combination of IR radiation and lab-on-a-chip technology [61]. Figure 12 presents an example of a pseudo-continuous flow FTIR system integrated on a microfluidic device for sugar identification [61]. Furthermore, the literature has already reported other miniaturised systems based on μFTIR for biological applications.

A study reported by G. Birarda et al. [62] demonstrated a protocol to build a low-cost IR-Live microfluidic chip for real-time 2D infrared imaging of living cells or tissues with a resolution in the range of micrometres. In this study, FTIR compatible microfluidic chips were produced by direct photolithography of a resist layer coated onto one large IR window (40 mm diameter), with an inlet connected to a tubing system and an outlet attached to a circular reservoir [62]. In the centre of the device, there is an IR-transparent experimental chamber sandwiched between two CaF_2_ crystal discs. The results of IR imaging on migrating cells with the subcellular spatial resolution can distinguish different cellular organelles and identify their peculiar chemical composition at a functional group level. The authors, through the performed assays (n = 14), were able to show the characteristic shapes of the proteins (amide II bands) and lipids (CH_2_-CH_3_ stretching) in the cells. The spectrum has a sharp protein signal centred at 1654 cm^−1^, mainly attributed to an α-helix protein structure [62].

Another method was developed for rapid ATR-FTIR monitoring solute concentrations in solutions flowing through microchannels [63]. The method involves the interface of commercially available ATR-FTIR instrumentation with a customised microfluidic device, which is sufficiently robust to withstand flow rates of the liquids of at least 20 mL h^−1^. The authors reported that the paper opened the way for on-chip identification of chemical compounds, measurements of their concentrations in solutions, and studies of reaction kinetics. Furthermore, the method can be used to characterise the adsorption of chemical and biological species adsorbed on the ATR surface under flow. From the spectrum in the region of 1400 cm^−1^–900 cm^−1^, the authors reported peaks at 1100 cm^−1^ and 1250 cm^−1^, which correspond to the antisymmetric and symmetric vibrational modes of the COC groups, respectively, and a peek at 950 cm^−1^, which is from the C=C bonds in its phenyl ring. The authors also focused on the dominant band at 1100 cm^−1^ and plotted the variation of its absorbance vs. concentration of TX-100 (*C*TX-100) in the solution. For *C*TX-100 ≥ 5 mM, the absorbance of the band linearly increased with the increasing solute concentration. According to the authors, the method allows the rapid acquisition of spectra and enables chemical characterisation and concentration measurements independent of the flow rate of liquids. The method enables the independent measurement of concentrations of solutes with distinct spectral features in mixed solutions. For the polymer solutes, the authors report that the method has a sensitivity of at least 10 µM (0.01 wt%). The authors also proposed the method’s applicability for the differentiation between dissolved and adsorbed amphiphilic species [63].

## 5. Conclusions

In this review paper, among the various spectroscopic techniques developed, FTIR is presented as a technique with the potential for distinguishing healthy from pathological samples. Numerous works have used FTIR with other techniques, such as ATR or micro-FTIR, to improve and simplify the spectral result of FTIR spectroscopy. The ATR-FTIR method promises the potential for the study of cells and tissues in general and, in particular, as a tool for estimating the metastatic potential of cancer cells. ATR-FTIR spectroscopy was also able to accurately identify HIV-infected pregnant women based on blood plasma, demonstrating the potential of this method for early detection of HIV in a rapid and reagent-free approach.

Label-free FTIR spectroscopy allows greater accuracy and reproducibility in cancer diagnosis while eliminating the need for complex and time-consuming clinical processing of tissue samples, currently required by existing computerised histopathological diagnosis. In addition, FTIR spectroscopy has also shown the potential to rapidly and objectively evaluate surgical resection margins to aid in surgical decision making, which may improve long-term survival and postoperative patient recovery compared with standard intraoperative pathological examination.

FTIR has also been used to monitor the response to cancer treatments and follow-up patients for treatment planning, early detection of recurrence, and assistance with psychological or psychosocial distress, with results that are faster, more sensitive, and more specific than conventional methods. Therefore, FTIR spectroscopy would be crucial to accelerate point-of-care decisions and potentially revolutionise cancer diagnostics in personalised medicine.

FTIR is the future measurement technique that shows tremendous potential and effective solutions to a large number of diagnostic complexities now faced by medical professionals. For instance, due to the limitations of the current gold standard techniques, FTIR may be advantageous to distinguish between normal samples and cancerous samples at an early stage, which offers the chance to diagnose and treat samples before any symptoms appear in patients. All these advantages force the researchers to dive deep into FTIR technology to move from a recognised to a viable technique used in the biological field, as in the other fields (environmental and chemical engineering, for instance) that are already recruiting FTIR for several applications.

## 6. Future Trends

Although FTIR is in continuous development in the biology field, the number of studies focusing on topics within the FTIR framework is steadily growing. In the future, FTIR may have a significant impact on various aspects of the medical field (i.e., hospital design, lab technician practices), including the financial. FTIR might bypass much equipment currently in use, as well as a large number of reagents used to perform the blood tests, thus proving to be a fast, convenient, economical, practical, and accurate method with high-quality results and minor environmental impact. However, to the best of the authors’ knowledge, despite the great interest of the scientific community in FTIR, there are only a few microdevice platforms reported in the literature. Lab-on-a-chip devices, with integrated ATR-FTIR measurements for medical applications in real-time label-free living biological systems analysis, deal with the problem of water presence, either using cell culture medium, plasma, or serum samples, once the absorption values could overlap the bands of other components [64]. In this perspective, ATR-FTIR is the best option to be integrated to study both hydrated and dried biological samples, such as cells and fluid flow [64]. Specifically, in the field of biomechanics and living mechanobiology, ATR-FTIR spectroscopy visualisation and quantification have also been demonstrated to be an excellent method for nondestructive biological analysis. Apart from all the cancer diagnostics biomarkers discussed in this work, cells’ alterations related to diseases occur in most blood pathologies associated with mechanical and rheological changes. The detection and quantification of mechanical alterations have hundreds of applications in diverse fields, ranging from the analysis of cell biomechanics to the classification of tissue biopsies [64,65,66]. For example, mechanical differences in exosomes and microvesicles reflect changes in cell biomechanics and the cell type, state, treatment, and phenotype [66]. Thus, their quantification and analysis are important for diseases stratification and personalised medicine, showing ATR-FTIR as an advantageous strategy.

Another example is the RBC deformability analysis that is affected by several factors, such as ageing, high blood sugar levels, total cholesterol, or functional oxidative stress. Thus, their membrane and internal cytoplasm suffer changes which biochemical analysis (particularly ATR-FTIR) with morphological and rheological techniques can define and then provide a profile indicative of deformability alteration [67]. As ATR-FTIR technology for blood or body fluids analysis requires proper sample preparation, integration of microfluidics can play, once more, an important role in the development of strategies for sample preparation, such as cells or plasma separation devices, single-cell sorting, cell deformability devices, droplet generators, and cell traps, among others. It is known that spectral analysis demonstrated that deeply deformed cells have different cellular biochemistry compared to nondeformed ones. So, it is expected that significant improvements can be obtained by integrating sample preparation microfluidic systems, enabling the RBCs, white blood cells, or circulating tumour cells analysis, in terms of their membrane biochemical quantification and consequently their biomechanical behaviour [68]. Looking forward to applications in tumour-on-a-chip devices, transparent 3D microfluidic devices will allow ATR-FTIR microspectroscopy applications to monitor the biochemical response to both mechanical and chemical stimulations (i.e., drug resistance).

The development of such devices will be a step ahead in state of the art and will overcome the limitations of current technologies. Thus, to achieve such a goal, future works need to consider the design, fabrication, characterisation, and optimisation of lab-on-a-chip platforms, with IR radiation and Fourier Transform postprocessing, to examine blood cells, distinguishing between normal and pathological ones, and to better understand several mechanisms of treatment resistance and progression.

## Figures and Tables

**Table 1:** InfraRed Regions [22,23].

Region	Wavelength (µm)	Wavenumbers (cm^−1^)	Frequency (×10^14^ Hz)
Near-IR	0.8–2.5	12,500–4000	3.75–1.2
Mid-IR	2.5–25	4000–400	1.2–0.12
Far-IR	25–100	400–100	0.12–0.03
Frequently Used	2.5–15	4000–670	1.2–0.20

**Table 2:** Characteristic FTIR spectral data of human blood antigens (a–antigen) for blood grouping applications [44]. Reprinted with permission from the authors and the International Journal of Science, Environment and Technology.

a				Functional Groups
A	B	AB	O	
1166	1166	1166	1163	Fucose linked to galactose via glycosidic linkage
1022	1020	1022	1020	Fucose residues linked to GlcNAc via glycosidic linkage
1022	-	1022	-	GalNAc glycosidically bonded to O antigen
-	1166	1166	-	Additional Galactose glycosidically bonded to O antigen

**Table 3:** Examples of applications of FTIR in the biological field.

Authors	FTIR Technique	Sample	Analytes	Application	Ref.
L. M. Rodrigues et al.	micro-FTIR	lesions and normal oral mucosa	collagen, lipids, fat acids, proteins, and amino acids	Evaluation of inflammatory	[46]
M. Pachetti et al.	ATR-FTIR	sperm	Proteins (α-helix, β-structures) and lipids	Reveal Lipid and Protein Changes Induced on Sperm by Capacitation	[47]
S. HamanBayarı et al.	ATR-FTIR	archaeological bone	carbonation of a phosphate	discrimination of human bone remains	[48]
A. Rutter et al.	FTIR	peripheral blood mononuclear cells, a leukaemia cell line, and a lung cancer cell line	lipids	Identification of a Glass Substrate to Study Cells	[49]
R. Minnes et al.	ATR-FTIR	mouse and human melanoma cells	amide II	distinguish between melanoma cells with a different metastatic potential	[50]
M. Polakovs et al.	EPR and FTIR	blood	*g*-factor In Methemoglobin	Study of Human Blood after Irradiation	[51]
P. Zarnowiec et al.	FTIR	human bacteria	Protein	Identification and Differentiation of Pathogenic Bacteria	[52]
M. J. Baker et al.	FTIR	blood	lipids, proteins, carbohydrate, and nucleic acids	Analyse biological materials	[53]
S. Mordechai et al.	FTIR	white blood cells (WBCs) and plasma	Protein and amino acids	Early diagnosis of Alzheimer’s disease	[54]
M. Martin et al.	ATR-FTIR	plasma and whole blood	proteins, nucleic acids, lipids, and carbohydrates	The effect of common anticoagulants in detection and quantification of malaria parasitemia in human red blood cells	[55]
I. C. C. Ferreira et al.	ATR-FTIR	saliva	proteins, nucleic acids, lipids, and carbohydrates	Analysis of Saliva for Breast Cancer Diagnosis	[56]
C. Aksoy et al.	FTIR spectroscopy and imaging	stem cells	lipids, proteins, glycogen, and nucleic acids	Effect of the donor age on human bone marrow mesenchymal stem cells	[57]
V. Shapaval et al.	FTIR	food-related fungal strains cultures	fungi detection through protein quantification	Characterisation of food spoilage fungi	[58]
G. Güler et al.	ATR-FTIR	prostate cancer stem cells	Protein, nucleic acid, lipid, and carbohydrate	CD133+/ CD44+ human prostate cancer stem cells	[59]

## Data Availability

Not applicable.
